# *Exophiala angulospora* infection in hatchery‐reared lumpfish (*Cyclopterus lumpus*) broodstock

**DOI:** 10.1111/jfd.12940

**Published:** 2019-01-11

**Authors:** Marcia Saraiva, Max J. Beckmann, Sara Pflaum, Marianne Pearson, Daniel Carcajona, James W. Treasurer, Pieter van West

**Affiliations:** ^1^ Aberdeen Oomycete Laboratory, International Centre for Aquaculture Research and Development Institute of Medical Sciences Foresterhill, Aberdeen UK; ^2^ Fish Vet Group Inverness UK; ^3^ FAI Aquaculture Ardtoe Marine Research Facility Acharacle UK

**Keywords:** cleaner fish, *Cyclopterus lumpus*, *Exophiala angulospora*, Fungus, lumpfish

## Abstract

Samples from moribund lumpfish were collected in a marine hatchery in Scotland in 2015. Black nodules were noted on the skin, and gills and fungal hyphae were extensively distributed in musculature and internal organs. Multifocal chronic inflammatory lesions displaced structures in all affected organs. Mortalities commenced on completion of spawning in May and were evenly distributed over the second year in the temperature range 11–15°C. The main systemic infection causing agent was initially identified based on morphological characteristics as an *Exophiala* species. Ribosomal DNA (rDNA) ITS regions of the isolates were subsequently sequenced confirming the isolates belonged to *Exophiala* genus*. *All isolates fell in a single phylogenetic cluster, which is represented by *Exophiala angulospora*. Fish were treated with either formalin or Bronopol or a combination of both, but there was no effect on the pattern or numbers of mortalities. Isolates were also tested against three different concentrations of Latrunculin A, Amphotericin B and Itraconazole with no success. It is of utmost importance to increase the knowledge on pathogen–host interactions to successfully develop sustainable control methods.

## 1 INTRODUCTION

One of the major health problems that the Atlantic salmon (*Salmo salar*) industry faces at the sea farming stage is the sea louse, *Lepeophtheirus salmonis* Krøyer. This ectoparasite attaches to fish skin and mucosa (Boxaspen, 2006) inducing lesions that lead to a loss of body fluid and can be a point of entry for secondary pathogens and stress that may result in fish mortality if untreated (Denholm et al., 2002; Johansen et al., 2011). There is great interest in deploying lumpfish *Cyclopterus lumpus* as cleaner fish to control sea lice biologically on farmed salmonids in Europe and Canada (Powell et al., 2017). Lumpfish rearing commenced in 2014 using eggs from wild‐caught fish in Norway, Iceland, Ireland, Scotland and more recently in Canada. The lumpfish is the only member of the Cyclopteridae family and has a rounded body, thick skin and tubercles. Lumpfish is commonly found in north temperate coasts in the Bay of Biscay, the British Isles, Norway, Greenland and Canada (Davenport, 1985). The fishery is mainly near Iceland, Norway and Canada, and the annual catch increased to 20,365 tonnes in 2013 (FAO, 2017). The eggs are shed in a tight benthic clump of around 100,000 eggs per batch. These hatch after 270–300 degree days (dd) and the well‐developed larvae are large at ca 5 mm length and initiate external feeding within 1–2 days (Davenport, 1985). Live feed is the first feed of many marine finfish larvae but *Artemia nauplii* are only required briefly in lumpfish farming before transition to dry formulated feed, and many hatcheries have eliminated live feed altogether. The broodstock mature for the first time in the wild at ages of 3–6 years although, with rapid growth rate, fish can spawn in hatcheries from 1 year old. Most lumpfish eggs have been obtained by catching fish from the wild and stripping them on receipt in the hatchery. This has given good results but there is concern for wild stocks, about possible biosecurity risks of sourcing eggs from wild‐caught fish, and there has been a desire to move to use broodstock from hatchery reared production (Jonassen, Lein, & Nytrø, 2018; Wittwer & Treasurer, 2018). This will minimize the requirement for wild stocks, allow access to disease free, established photoperiod controlled stocks to enable year‐round production and also to permit the initiation of breeding programs to select for disease resistant fish and primarily to identify families with high levels of cleaning activity.

Several diseases have been reported in lumpfish. They are susceptible to bacterial diseases such as Vibrios, atypical furunculosis and Pasteurella (Powell et al., 2017, Scholz, Glosvik, & Marcos‐López, 2018) and the parasites *Paramoeba perurans, *the agent of amoebic gill disease AGD, and *Gyrodactylus* sp. (Alarcón et al., 2016). Fungus infection of lumpfish has been reported from Scotland (Powell et al., 2017) and also from Ireland and Norway (Scholz et al., 2018). Fish with similar symptoms have been reported in the Faroes (Johannesen, Arge, & Eliasen, 2018). There does not appear to be an effective treatment for fungus infection (Powell et al., 2017; Scholz et al., 2018). Fungal mycoses in fish are common but most affect external tissues with few species found in internal organs (Verma, 2008).

These reports identified the main agent of mycosis in lumpfish as *Exophiala* sp. The *Exophiala* genus is an anamorph genus from the Ascomycete family and *Chaetothyriales *order (Gjessing, Davey, Kvellestad, & Vrålstad, 2011). This genus consists of several potential opportunistic/pathogens that take advantage of immunocompromised organisms. The most serious pathogen, even capable of resulting in human mortality, is *Exophiala dermatitidis *(Sudhadham et al., 2008). Nevertheless, the *Exophiala* genus has been described as deadly to several fish for the past decades, and amphibian and aquatic animals in general (Gjessing et al., 2011; de Hoog et al., 2011). *Exophiala salmonis*, *Exophiala psychrophila *and *Exophiala pisciphila* are usually the major agents. *Exophiala* sp. has rarely been found in juvenile lumpfish to date before they are transferred to sea cages. There have also been no reports of *Exophiala* sp. having been transferred to salmon. Another species *E. salmonis* (=*E. psychrophila*) was reported in salmon by Richards, Holliman, and Helgason (1978) but this appears to be a rare occurrence in salmon and the infection in that case was confined to the kidney. However, it became evident that some hatchery‐reared lumpfish broodstock were showing clinical signs of an unidentified fungus infection, and that species identification, an understanding of the pattern of infection, and some form of control method were required. Nevertheless, due to the lack of knowledge, it is difficult to accurately estimate the magnitude of the problem. The current work examines the clinical signs of disease, identification of the fungus, epidemiology of infection and also means of treatment and management of *Exophiala angulospora* infection.

## 2 MATERIALS AND METHODS

### 2.1 Lumpsucker origin and maintenance

Lumpfish were reared from eggs stripped from wild fish caught in the English Channel in 2014. Fish were held in containers in the sea and stripped and hand fertilized within 5 days of collection. Eggs were incubated for 48 hr at 9°C and then shipped to a marine hatchery in Scotland where they hatched from 270 to 300 degree days (dd). Fish were stocked in 1,300 L volume black polypropylene tanks. Larvae were fed initially on *Artemia* and then weaned to dry feed after 10 days, Biomar Prowean followed by Biomar Inicio. Fish grew to over 20 g in 6 months using a flow‐through rearing system. The 50 largest fish were selected from each of three tanks with fish from different parents and maintained in circular polypropylene 1,600 L tanks until February 2015 when the lumpfish were transferred to outside circular tanks of 2 m depth and a volume of 28 m^3^.

### 2.2 Infection monitoring and treatment

Mortalities due to fungus infection commenced when fish were 14 months old and immediately after the spawning season was completed. Fish were identified with black nodules in several external locations and were culled and sampled. Samples from skin and internal organs, heart, liver, ovary, kidney, gut and cecae were collected. Samples of fish feed were taken and tested for spoilage with fungus species. The losses of fish to various agents were recorded over 1 year, and data were examined for any pattern in mortality to fungus. The observed frequency distribution was compared with what would be expected if the data were normally distributed and analysed for goodness‐of‐fit with a chi‐square test. The broodstock lumpfish in the three tanks were treated for fungus infection on ten occasions with either 200 ppm formalin for 1 hr or 40 ppm Pyceze and latterly with a combination of both medicines together at 3‐day intervals over a 3 treatment cycle.

### 2.3 Isolates purification and culturing

Upon arrival to the Aberdeen laboratory, the fish tissues were divided further and placed on multiple media plates: Potato dextrose agar (PDA) with and without salt, Potato dextrose agar with fish peptone; Sabouraud dextrose agar (SDA) with and without salt and blood agar. All media were supplemented with vancomycin (100 mg/L), ampicillin (500 mg/L) and chloramphenicol (10 mg/L) and incubated at 12°C until mycelial growth was clearly visible and could be excised. The plates were further cleaned up to produce axenic isolates. Once axenic, isolates were re‐inoculated into PDA and sub‐cultured at 12°C.

### 2.4 DNA extraction

Genomic DNA from the axenic cultures was extracted following the protocol previously described by Zelaya‐Molina, Ortega, and Dorrance (2011). The DNA was cleaned from other nucleic acids by addition of 1 µl of RNase A (20 mg/ml, Sigma‐Aldrich) and incubation for 30 min at 37°C. The quality of the DNA was checked spectroscopically (NanoDrop) and quality visually assessed on 1% (w/v) agarose gels.

### 2.5 Isolates identification

All isolates were identified by PCR and subsequent sequencing reaction of the internal transcribed spacer (ITS) region 1 and 2 including the 5.8 s rDNA region. For the ITS PCR and sequencing reaction, the following primers were used as described in White, Bruns, Lee, and Taylor (1990):

ITS4: 5′‐TCCTCCGCTTATTGATATGC‐3′.

ITS5: 5′‐GGAAGTAAAAGTCGTAACAAGG‐3′.

The PCR reaction was performed in 25 μl reaction mix containing 5 μl of 5 × colourless flexi buffer (Promega, UK), 10.75 μl nuclease‐free water, 5 mM Mg_2_Cl_2 _(20 mM), 0.2 mM dNTPs (10 mM), 0.4 μM of each primers (10 μM) and 1.25 units Go*Taq G2 *Polymerase (5 u/µl*, *Promega) and 1 μl of DNA template (~50 ng/μl).

The PCR reactions were run on a thermal cycler: 1 cycle of initial denaturation (95°C for 5 min), amplification for 30 cycles (95°C for 30 s, 57°C for 1 min and 73°C for 1 min 30 s) and finally 1 cycle of final extension (73°C for 7 min). The PCR products were separated by electrophoresis in 1.5% (w/v) agarose gels containing ethidium bromide.

### 2.6 Phylogenetic analysis

The ITS amplicons were sent to a commercial sequencing facility (Source Biosciences, Germany). The generated sequences were compared with other fungal ITS sequences from the GenBank sequence database using a BLASTN search algorithm. Using the software Molecular Evolutionary Genetics Analysis version 7 (MEGA7) (Kumar, Stecher, & Tamura, 2016), a data set was compiled of ITS nucleotide sequences of *Exophiala *spp. (34 obtained from GenBank), and a sequence alignment was subsequently performed using the ClustalW algorithm. Neighbor‐joining method was used to construct a bootstrap consensus tree from 1,000 replicates to determine the evolutionary history of the data set. Evolutionary distances were computed using the maximum composite likelihood method, and all ambiguous positions were removed for each sequence pair during analysis.

### 2.7 Histology

Tissue samples from gills, skin and skeletal muscle and visceral organs were fixed in 10% (w/v) buffered formalin, embedded in paraffin wax blocks, sectioned (4 and 6 μm thick) and stained with hematoxylin and eosin, periodic schiff (PAS) briefly. Sections were first washed with water and then a 1% (v/v) aqueous solution of periodic acid was applied for 15 min. The sections were washed to remove periodic acid in excess, and then, the Schiff's reagent was applied for 10 min. The Schiff's reagent was rinsed off with water; afterwards, the sections were washed with water until the water was clear and the sections were pink, usually about 10–30 min. Fungal hyphae were stained using Grocott's methenamine silver stain (GMS) (Grocott, 1955). Images were taken with the EVOS transmitted light imaging system (AMG, Washington, USA).

### 2.8 Antifungal resistance test

*Exophiala* isolates (ABDN001Ea, ABDN002Ea and ABDN003Ea) were tested against three different antifungals (Latrunculin A, Amphotericin B and Itraconazole) in three different concentrations (25, 50, 75 mg/L). PDA plates were prepared, and *Exophiala* isolates were inoculated and incubated at 12°C for 2 weeks. In aseptic conditions, discs containing the different antifungal concentrations were placed 1 cm from the colony and 3 cm from each other. Three replicates per antifungal and per concentration were analysed.

## 3 RESULTS

### 3.1 Clinical infection and pathology

**Figure 1:**
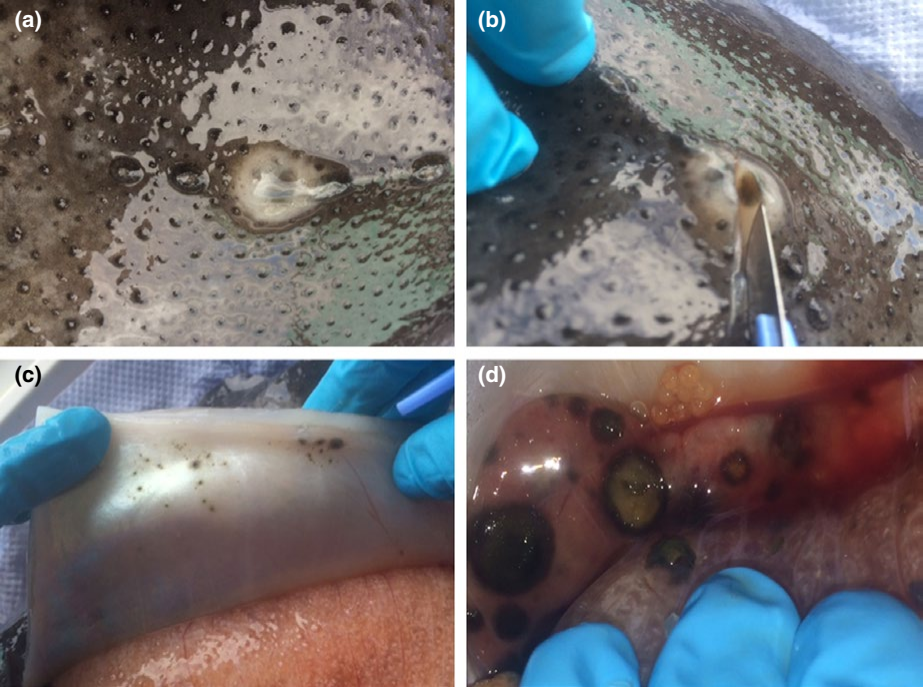
Lumpsucker showing multifocal black areas scattered across internal organs. Panel a: external lesions, Panel b: showing in depth one external lesion, Panel c: internal muscle and Panel d: kidney. Lesions were excised, and axenic cultures were obtained and further identified. Pictures provided by Sara Pflaum (Fish Vet)

Lumpfish were first noticed, at an age of 12 months, with dark lesions and suspected fungal infection in May 2015 (Figure 1a, b). Lumpfish did not show signs of morbidity, nor diminished swimming activity. Multifocal black areas scattered across all internal organs, in particular the kidney (Figure 1d), were apparent with many of the infected fish showing external skin lesions (Figure 1a, b). The appearance of these external lesions was different in terms of colour and structure. However, many of these external lesions had black areas at their core and corresponded to black areas on the internal body wall (Figure 1b). Fungal hyphae were readily visible in wet preparations examined at ×100 magnification and later in histology. Lesions, often circular and in the range 2–14 mm diameter, were present on the body surface and more visibly on the belly and the gills.

### 3.2 Histology

**Figure 2:**
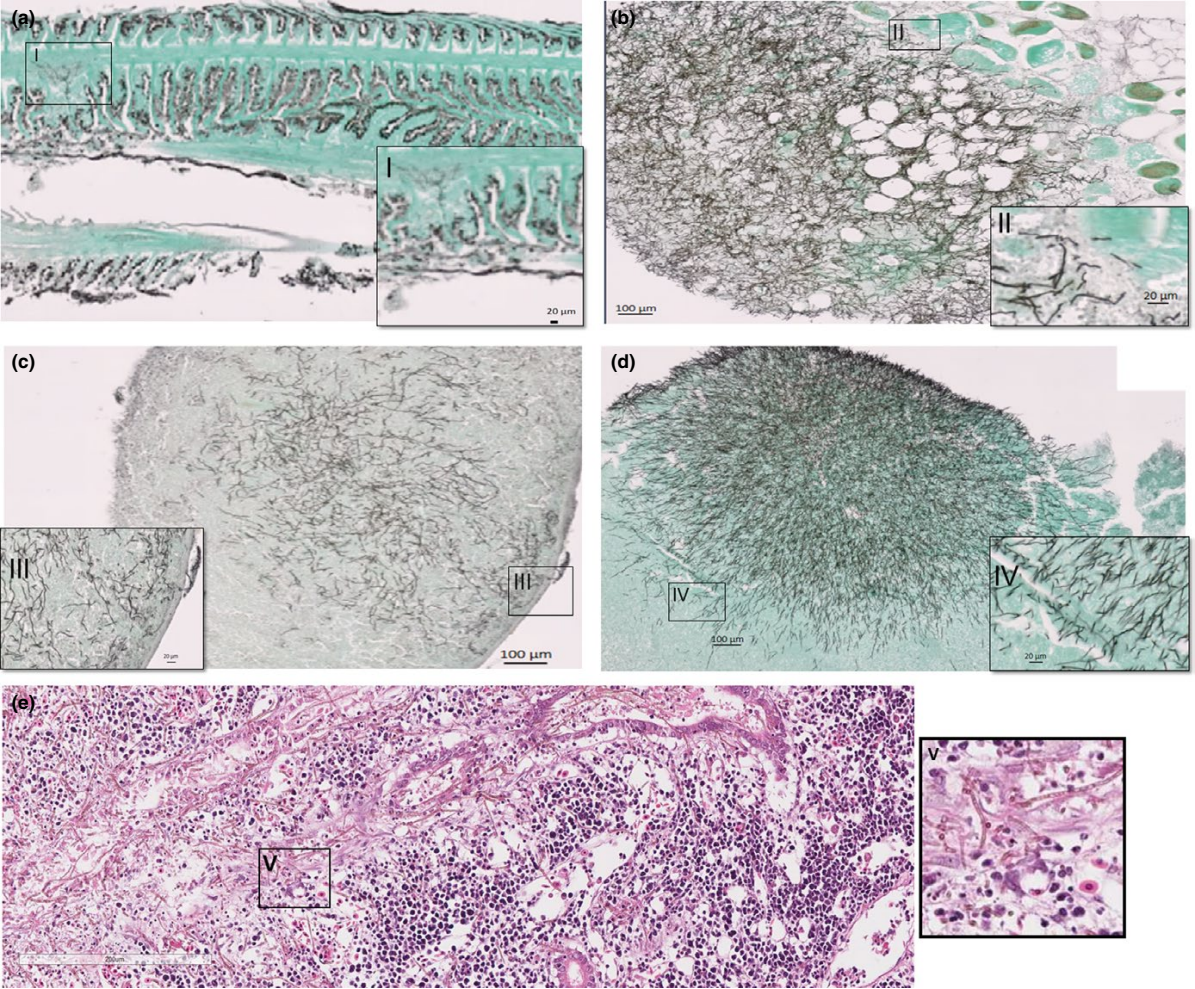
Lumpfish organ tissue microscopy. Different organs tissue were processed and analysed for the presence of fungi. PAS/GMS staining. Panel a—gill tissue, Panel b—muscle tissue, Panel c—spleen tissue, Panel d—liver tissue. Fungus was found to grow in most internal organs with more prevalence in the kidney (Panel e)

Fungal hyphae were extensively distributed in the body musculature and in all organs including liver, spleen, heart, kidney, gut and ovaries/testes. In histopathology, infection was reported as severe degenerative changes associated with large clumps of fungal hyphae displacing gill filaments (Figure 2a). Debris and bacteria were embedded in the surface of the mycelium. Smaller multifocal areas of haemorrhage, degeneration and inflammation were associated with fungal hyphae arising from blood vessels suggesting haematogenous spread. Degenerative changes were evident in the skin associated with fungus throughout the epidermis and dermis and invading underlying muscle. Multifocal chronic inflammatory lesions with fungal hyphae were present throughout the muscle (Figure 2b). Massive fungal mycelia displaced the majority of structures in the kidney (Figure 2e). Fungal hyphae were also identified throughout the ventricle and epicardium, and there were also small bacterial colonies in the myocardium. In other fish, there were multiple large chronic inflammatory lesions with fungal hyphae diffusely throughout in the heart, and small focal bacterial colonies were associated with some fungal inflammatory lesions. Massive fungal mycelium displaced the majority of stroma in the spleen (Figure 2c). Although all internal organs presented lesions, the kidney was found to be heavily infected. This could possibly indicate that the infections originated from the urinary tract. However, external lesions could also provide a point of entry for the fungus.

*Exophiala angulospora* strains have been isolated from water, decorticated wood, human skin and nails, soils and diseased fish (Gjessing et al., 2011; de Hoog et al., 2011). Due to the ubiquitous nature of the fungus, the origin of the *E. angulospora* inoculum found in the diseased lumpfish is unclear. However, it was not our aim to identify the pathogen source.

### 3.3 Species and strain identification

**Figure 3:**
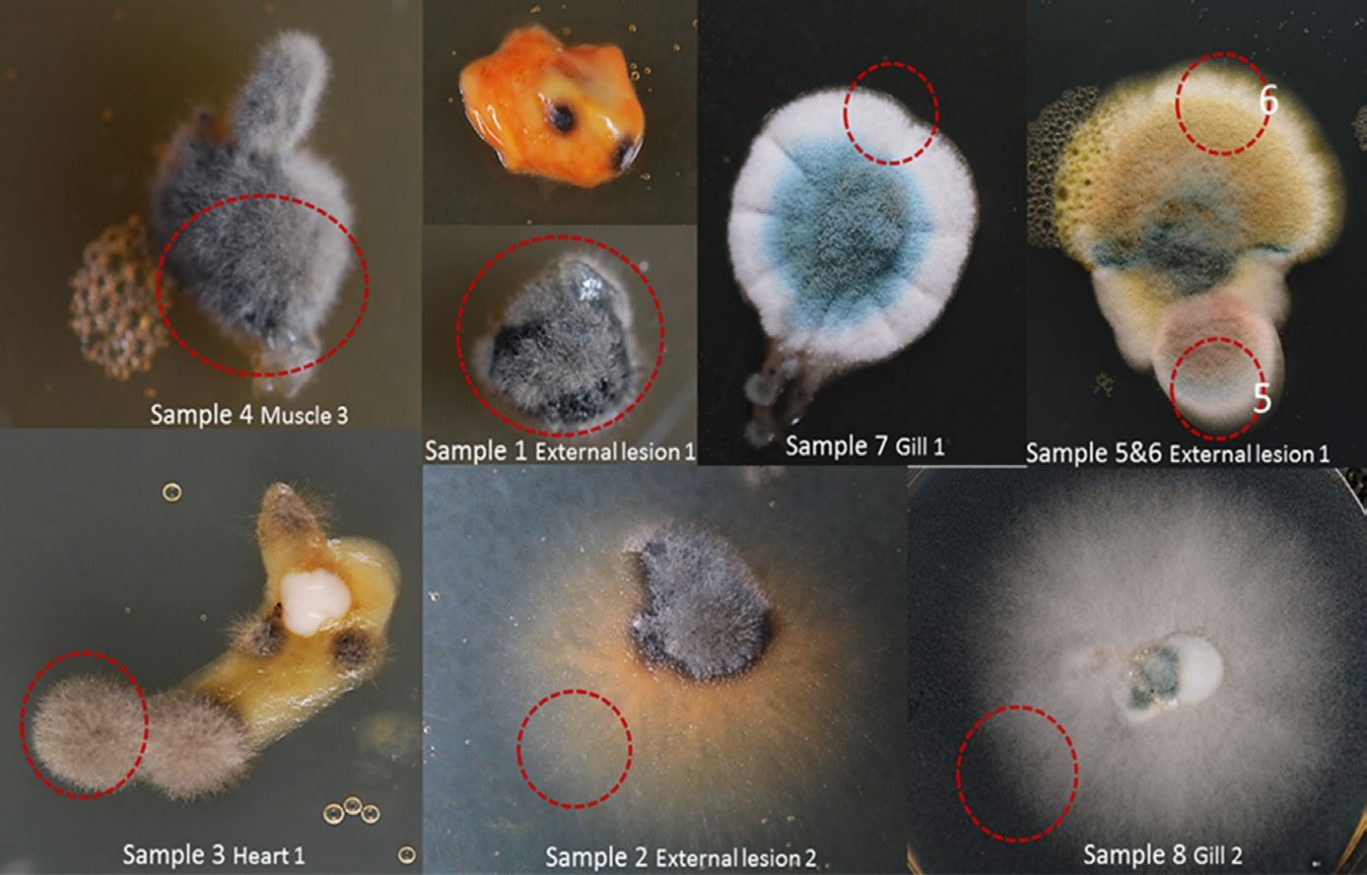
Fungus diversity found on lumpfish samples. Isolates were re‐inoculated on PDA supplemented with antibiotics until axenic cultures obtained. Two types of morphology can be observed—a mouldy and a penicillium‐like type. Further processing identified four different genera, *Exophiala*, *Emericella*, *Penicillium* and *Fusarium*. Red circles represent the colony piece that was excised for further processing

Examination of broodstock lumpfish that had been maintained on site for over a year indicated fungal infection due to the multifocal dark lesions on the surface of the fish and through the musculature. Fungal hyphae were also seen in fresh samples in microscopy at ×100 magnification and in histology. After 1 week, there was a grey and black velvet growth on multiple media slides. All tissue samples resulted in fungus isolates. These grew on potato dextrose agar although at different growth rates. The majority of isolates were slow growers on any media and temperature tested. 12°C was used to mimic environmental conditions. After 1 week of incubation, two morphology types could be clearly differentiated: a grey/black velvety growth or a mouldy penicillium‐like growth. The first morphology type was found in all tissue samples while the second type, penicillium‐like morphology, was only seen on gill and external lesion samples (Figure 3). Three different tissues samples presenting the grey/black velvety morphology and 5 from the mouldy type were selected for further analysis.

ITS PCR revealed 4 different band sizes clustering the samples into 4 possible groups. Sequencing of the combined ITS1, 5.8 s and ITS2 region and BLAST produced the following results: isolates 1, 3, 4 showed homology with *Exophiala *sp., isolate 2 showed highest homology with *Emericellopsis pallida*, *Emericella nidulans *and *Acremonium zonatum*, isolates 5,6,7 belong to *Penicillium* genus and isolate 8 showed homology with *Fusarium *sp.

**Figure 4:**
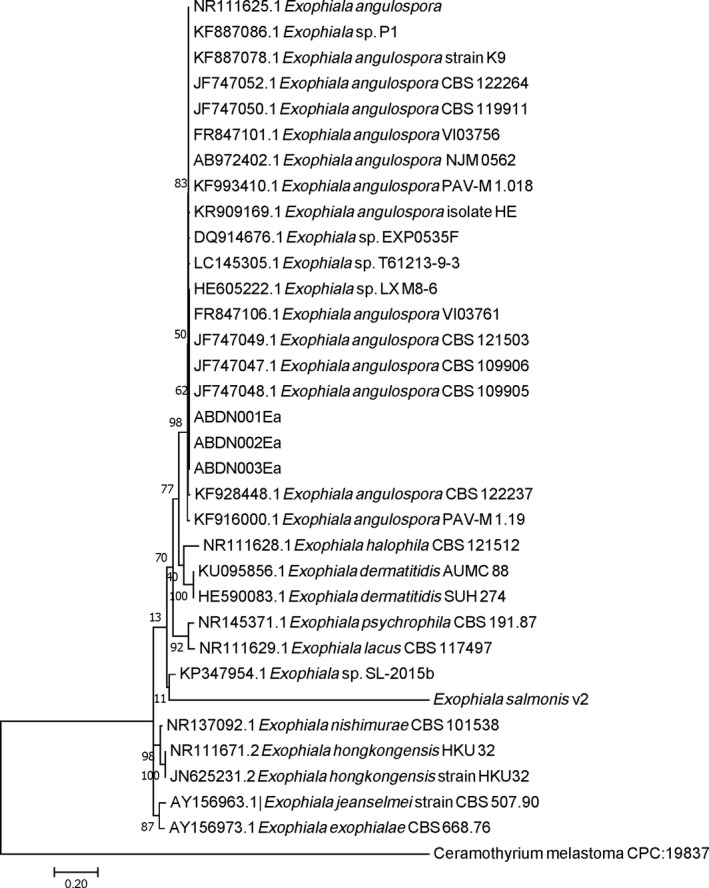
The bootstrap consensus tree inferred from 1,000 replicates using the Neighbor‐joining method based on ITS rDNA sequences from *Exophiala* species. The percentage of replicate trees in which the associated taxa clustered together in the bootstrap test (1,000 replicates) is shown next to the branches. The evolutionary distances were computed using the Maximum Composite Likelihood method (Tamura, Nei, & Kumar, 2004). The analysis involved 37 nucleotide sequences. All positions containing gaps and missing data were eliminated. There were a total of 410 positions in the final data set. Evolutionary analyses were conducted in MEGA7 (Kumar et al., 2016)

Three dematiaceous fungal isolates were obtained from the internal organ samples (denoted as isolates ABDN001Ea, ABDN002Ea and ABDN003Ea). BLASTN ITS sequence searches against GenBank returned a 100% sequence homology of the three isolates to various isolates of *E. angulospora*. A data set of the ITS rDNA gene was compiled and analysed to infer the relative evolutionary history of isolates with other *Exophiala *species representatives (Figure 4).

### 3.4 Epidemiology of infection

**Figure 5:**
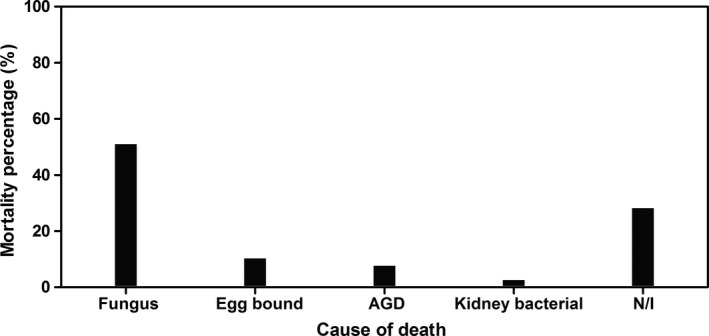
Categorization of lumpfish mortalities in their second year. Total mortalities *n* = 39, of 146 fish

**Figure 6:**
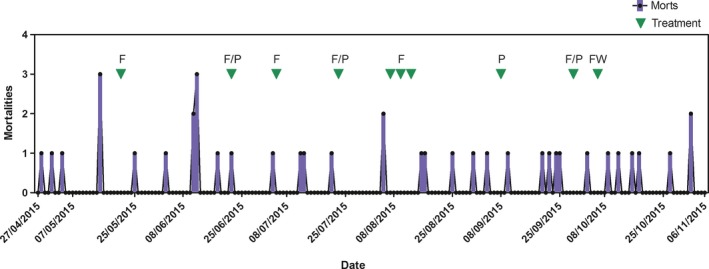
Lumpfish mortalities to all causes in their second year and treatment dates. Treatments for fungus were with either *F* = 200 ppm formalin alone; *F* and P = formalin and Pyceze 40 ppm; FW = freshwater bath for AGD

Mortalities in lumpfish from stocking as broodstock in October 2014 to spring 2015 were infrequent, with only 4 recorded losses from a total of 150 fish stocked, and there were no indications of fungus on autopsy or in wet preparations examined under the microscope at ×100. The first broodstock mortality in 2015 was reported on 28 April and was associated with fish being egg bound, and there were no nodules observed in the musculature. In total, there were 39 mortalities in 146 fish over the second year of the hatchery‐reared lumpfish. Fungus was the largest of the assigned causes of mortality with 20 fish (51%), followed by egg bound fish (10.3%), 7.7% were assigned as due to AGD, and 2.6% (1 fish) as a kidney bacterial infection, and in 11 fish (28.2%) the cause of mortality could not be identified (Figure 5). The first mortality to infection identified as *E. angulospora *was on 15 May at a water temperature of 11.2°C. Tanks were drained for routine inspection of fish, and three bloodstock lumpfish of mean male weight 480 g and female weight 1,140 g showed morbidity and external signs of fungal infection and were culled. The pattern of mortalities to *E. angulospora* infection which was detected in fish in all three broodstock tanks was thereafter regular through the summer, and the observed frequency was normally distributed (Chi^2^ = 95.9, *p* = 0.99). The highest temperature of 14.8°C was recorded on 27 August. Mortalities to fungus continued sporadically through the summer and autumn, and the last mortality attributed to fungus was on 2 November at a water temperature of 11.4°C (Figure 6).

Spawning commenced on 4 April and continued to 14 May, with a peak in April. There was therefore not a clear pattern of mortality or association in particular with spawning events, other than that mortalities commenced at the end of the spawning period when fish may have been immunocompromised, and mortalities also commenced as water temperature increased at the end of spring. Several thousand juvenile lumpfish were reared in 2015 from eggs obtained from wild fish captured in the south of England. These juvenile fish were handled for grading and also for vaccination, and no further incidence of fungal nodules was identified readily against the light green colour of the fish.

### 3.5 Treatments and management

The first three broodstock lumpfish exhibiting fungus nodules were culled on 15 May and treatment to contain the spread of the mycelia and to treat the remaining fish in which no overt infection was visible was carried out on 16 May with treatment with 200 ppm formalin for 1 hr (Figure 6). More lumpfish (*n* = 3) were noted with nodules on 16 June, and treatment with a combined 200 ppm formalin and 40 ppm Pyceze (50% w/v Bronopol) commenced on 19 June. Further mortalities to fungus occurred on 5 August, and combined formalin/Pyceze treatments were applied from 7 August and subsequently at 3‐day intervals (Figure 6). Mortalities continued after that date and more combined formalin/Pyceze treatments were applied at the end of September. However, losses to fungus continued at low levels in October and November. In total, 10 therapeutic bath treatment regimes were applied between May and November. There was no indication that the combined treatments were effective in preventing infection with *E. angulospora* nor in removing fungus from the environment and, inevitably, it was not possible to carry out continuous disinfection of tanks.

## 4 DISCUSSION

*Exophiala* spp. have been reported from a range of marine finfish (Řehulka, Kolařík, & Hubka, 2016). It has been suggested that the extent of host specificity is low and has extended beyond marine finfish to a range of cold‐blooded animals including amphibians, tortoises and crabs (de Hoog et al., 2011; Řehulka et al., 2016). More than 50 species of Exophiala have been recognized based on morphological and molecular genetic analyses. The physical distinction of *Exophiala* spp. is difficult due to an overlap in morphology. Identification has involved examination of growth, the temperature requirements and dimensions of conidia, together with DNA sequencing techniques (Řehulka et al., 2016). *E. angulospora* has been identified previously in lumpfish (Řehulka et al., 2016), and also in several other marine finfish such as cod *Gadus morhua* (Gjessing et al., 2011), halibut *H. hippoglossus* (Overy et al., 2015) and the Japanese flounder *Paralichthys olivaceus* (Kanchan, Muraosa, & Hatai, 2015). Another species of Exophiala, *E. psychrophila*, has also been diagnosed in lumpfish (Scholz et al., 2018). The lack of an effective treatment suggests that *Exophiala* species could be a major obstacle to broodstock selection programmes in lumpfish, unless a fungus‐resistant stock can be bred. In the current study, two of the fish species mentioned in the literature, cod and halibut, together with other marine finfish such as wrasse, salmon, turbot and sea bass have been stocked in adjacent tanks to lumpfish in the last few years with no appearance of *Exophiala* spp. This suggests that lumpfish may be particularly susceptible or prone to infection due to poor condition during and following spawning and issues with immune competence. To the best of our knowledge, *Exophiala* infection has not been reported in wild lumpfish.

Fungal infection will be an ongoing and widespread issue for maintenance of lumpfish broodstock. Areas where seawater passes through rock/lava and is filtered, such as in Iceland, may give protection against fungal diseases in the water supply. In the present study, broodstock were kept in the second year in outside tanks, rather than in the hatchery, and may have been susceptible to fungal infection from blown soil or other contaminants. There is no evidence of the origin of the fungal mycelium and it may be widespread in the environment, such as in soil with contamination by contact or being dispersed by air movements. *E. salmonis* (=*E. psychrophila*) has been reported in salmon in sea cages (Richards et al., 1978) and it was suggested that fish may have been infected by contaminated feed, and *E. salmonis* may be classified as a feed spoilage organism (Bruno et al., 1997). The feeds presented to the lumpfish in the present case were tested for spoilage, and there was no trace of *Exophiala* spp.

*Exophiala* infection has rarely been seen in juvenile lumpfish production in many millions of fish shipped to sea cages and, to date, has not been an issue in the hatchery phase. There has been no evidence of transfer of *Exophiala* from lumpfish to salmon in sea cages. Clinical infection with *Exophiala* was seen in lumpfish from mid‐May to November with losses being evenly distributed over this period and in all three tanks with broodfish. There did not appear to be an annual pattern in mortalities. Two associated factors affecting infection could be suggested. The first is an immunocompromised condition perhaps related to the spawning period and, in animals and humans, *Exophiala* infection is often associated with poor immune competence (Rimawi et al., 2013). The fish may have been exhausted and in poor condition at the end of the spawning period. There was an even distribution in mortalities in males and females and so there was no specific gender bias. Another possibility is that infection is simply a chronological event associated with the age of the fish, or related to periods of elevated water temperature above 10°C, as mortalities occurred from 15 May to 3 November when water temperature was in the range 11–15°C and it peaked in August, although this maximum temperature was not associated with a rise in mortalities.

Various treatments with only formalin and also in combination with Pyceze were assessed on various occasions but the pattern of lumpfish infection with *E. angulospora* here was not disrupted and it further did not appear to “recover” fish that may have already been infected with fungus. Also, three different antifungals were tested in vitro with no success. As there appears to be no form of successful treatment yet, the culling of broodfish may be the only possible action.

Future research is required in chemotherapy and medicinal use in preventing infection and in treatment of *Exophiala* spp. Although lumpfish have been found to be susceptible to a range of health issues including bacteria and parasites such as AGD these can be treated or protection can be afforded by vaccination in the case of *Vibrios*, furunculosis and *Pasteurella*. However, *Exophiala* infection may be a limiting factor for maintenance of own hatchery broodstock and in genetic selection programmes for traits such as cleaning ability, slow growth and resistance to diseases.

Quick and accurate diagnostic tools are urgently needed, not only for *Exophiala* species, but also for other fish pathogenic fungi and oomycetes in general, as outbreaks of mycosis in farmed fish and aquarium animals can cause severe losses (Sarowar et al., 2014). There are currently no approved antifungal agents in fish production in the United Kingdom.
